# Immunologic Predictors of Liver Transplantation Outcomes in HIV-HCV Co-Infected Persons

**DOI:** 10.1371/journal.pone.0135882

**Published:** 2015-08-27

**Authors:** Ashwin Balagopal, Burc Barin, Jeffrey Quinn, Rodney Rogers, Mark S. Sulkowski, Peter G. Stock

**Affiliations:** 1 Department of Medicine, Johns Hopkins University, Baltimore, MD, 21205, United States of America; 2 The EMMES Corporation, Rockville, MD, 20850, United States of America; 3 Department of Surgery, University of California San Francisco (UCSF), San Francisco, CA, 94122, United States of America; University of Toledo, UNITED STATES

## Abstract

Liver disease is a leading cause of mortality among HIV-infected persons in the highly active anti-retroviral therapy (HAART) era. Hepatitis C Virus (HCV) co-infection is prevalent in, and worsened by HIV; consequently many co-infected persons require liver transplantation (LT). Despite the need, post-LT outcomes are poor in co-infection. We examined predictors of outcomes post-LT. Immunologic biomarkers of immune activation, microbial translocation, and Th1/Th2 skewing were measured pre-LT in participants enrolled in a cohort of HIV infected persons requiring solid organ transplant (HIVTR). Predictive biomarkers were analyzed in Cox-proportional hazards models; multivariate models included known predictors of outcome and biomarkers from univariate analyses. Sixty-nine HIV-HCV co-infected persons with available pre-LT samples were tested: median (IQR) CD4+ T-cell count was 286 (210–429) cells mm^-3^; 6 (9%) had detectable HIV RNA. Median (IQR) follow-up was 2.1 (0.7–4.0) years, 29 (42%) people died, 35 (51%) had graft loss, 22 (32%) were treated for acute rejection, and 14 (20%) had severe recurrent HCV. In multivariate models, sCD14 levels were significantly lower in persons with graft loss post-LT (HR 0.10 [95%CI 0.02–0.68]). IL-10 levels were higher in persons with rejection (HR 2.10 [95%CI 1.01–4.34]). No markers predicted severe recurrent HCV. Monocyte activation pre-LT may be mechanistically linked to graft health in HIV-HCV co-infection.

## Introduction

Liver disease is a leading cause of mortality in HIV despite major advances in treatment with highly active antiretroviral therapy (HAART).[1] HIV-HCV co-infection occurs in one-third to 80% of HIV-infected persons.[2–4] Chronic HCV infection is the most common indication for liver transplant (LT) in the U.S., but complications post-transplant include acute rejection, graft loss, and severe recurrent HCV. Indeed, HIV-infected patients have worse outcomes after liver transplantation than HIV uninfected patients, although the underlying mechanisms are unknown.[5] Immune activation is thought to drive AIDS progression in the non-transplant HIV-infected population[6] and may contribute to liver disease progression; we found that microbial translocation (MT) from intestinal CD4+ T cell depletion was associated with immune activation and was strongly associated with cirrhosis in HIV-HCV co-infected persons.[7] Given the strong evidence that links immune activation and liver disease in animal models, we hypothesized that immune activation in HIV-HCV co-infected persons may drive transplantation outcomes.[8]

The Solid Organ Transplantation in HIV Multi-site Study (HIVTR, AI 052748) is a cohort of HIV-infected persons who have been followed before and after organ transplantation to examine long-term graft and individual survival. Earlier research in this cohort has shown that HIV-infected LT candidates have higher mortality pre-LT than HCV mono-infected controls, and that this mortality was explained on the basis of MELD scores.[5, 9] Surprisingly, HIV-infected persons had nearly six-fold higher mortality than HIV-uninfected controls even at low MELD scores (15–19) after adjustment for CD4+ T cell count and HIV RNA. These results suggest that pre-LT determinants may influence outcomes post-LT, although the mechanism(s) underlying early mortality are not known. The present study was designed to identify baseline immunologic correlates pre-LT that predicted post-LT outcome. Immunologic phenotypes were broadly divided between signature markers of MT, innate immune activation, and adaptive T cell function; Th1/Th2 balance was addressed in the latter category. In addition, IP-10 was measured pre-LT as a chemokine that is highly predictive of liver disease progression in chronic HCV infection.

## Methods

### Study subjects

The HIVTR study enrolled HIV-infected subjects with evidence of end-stage liver disease (ESLD) who were candidates for liver or liver-kidney transplantation at 17 transplant centers in the United States between October 2003 and February 2010 (clinicaltrials.gov, NCT00074386). Eligible patients met site criteria for placement on the liver transplant wait-list. In addition, patients were only included with CD4+ T cell counts > 100 cells μL^-1^, or > 200 cells μL^-1^ if there was a history of prior opportunistic infections. Similarly, patients were only included with HIV RNA < 75 cp mL^-1^; exceptions were made for patients with HAART hepatotoxicity who were predicted to develop HIV virologic suppression with HAART post-LT. Additional exclusions were patients with a history of progressive multifocal leukoencephalopathy, chronic intestinal cryptosporidiosis, primary central nervous system lymphoma, multidrug-resistant fungal infections, or significant wasting. A total of 125 liver recipients were enrolled in the study, 89 of which were HCV-infected.

### Institutional Review Board Approval

The Committee on Human Research at the University of California, San Francisco approved the study protocol, as did the Internal Review Board from each center, as has been previously reported.[5] Each participant provided written informed consent for bloodbanking, testing, and analysis of samples.

Full immunologic profiling was performed on 69/89 HIV-HCV co-infected liver recipients with available pre-LT serum samples; 4 were also HBV-infected. Pre-LT samples were collected at a median (IQR) time of 16 (0–87) days prior to transplant; 93% of pre-LT samples were collected within 6 months prior to transplant and only 1 sample was collected > 1 year prior to transplant.

Liver biopsies were required at least annually to assess disease severity, were read centrally and scored using the Ludwig-Batts criteria to measure liver disease grade and stage. Additional liver biopsies were performed for liver enzyme abnormalities, and to confirm suspected graft rejection or drug toxicity. HAART was often discontinued at the time of transplantation due to toxicity as is conventional practice; HAART was reinitiated, usually within the first post-operative week, by an HIV provider in consultation with the transplantation team and in most instances subjects continued their pre-LT HAART regimens in the post-LT period. Use of immunosuppressive medications post-LT has been described previously.[5]

Cases were identified by graft loss, acute rejection, or severe HCV recurrence. Graft loss was defined by the need for a secondary transplant, and was identified histopathologically or clinically. Acute rejection was defined by the need for treatment with immunosuppressive medications above maintenance, as defined by each site. Severe HCV recurrence was defined histopathologically by cholestatic hepatitis, bridging fibrosis, or cirrhosis, or was defined clinically by graft loss due to HCV.

### Laboratory methods

Serum samples were aliquotted into 1 mL cryopreserved tubes and stored in -80°C freezers until testing. All laboratory testing was performed at one site. Lipopolysaccharide, an inflammatory cell wall component of Gram-negative bacteria, was tested using a modified *Limulus* Amebocyte Lysate assay, recently optimized for testing human specimens.[10] Commercially-available ELISA kits were used to measure sCD14 (R&D Systems, Minneapolis, MN), sCD163 (IQ Products, Groningen, Netherlands), IP-10 (R&D Systems, Minneapolis, Minnesota), and Neopterin (Brahms GmbH, Henningsdorf, Germany). The remainder of the analytes were measured using the Meso Scale Discover multiplex platform (MSD; Gaithersburg, MD) that has been previously described.[11] Briefly, each well of a 96-well plate is pre-coated with antibodies to TNFα, IL-1, IL-2, IL-4, IL-5, IL-8, IL-10, IL-12p70, and IFNγ. After incubation of serum into the relevant well for cytokine capture, electrochemiluminescent-labeled detection antibodies are bound to the cytokine and quantified using a charge-coupled device.

### Statistical analyses

Descriptive statistics included proportion, median and interquartile range (IQR) as appropriate. Comparison of baseline characteristics was conducted using the Fisher’s exact test (categorical variables) or Wilcoxon rank-sum test (continuous variables). Univariate and multivariate Cox proportional hazards models were constructed for graft loss, acute rejection, and severe HCV recurrence separately and as has been described previously.[5] Multivariate models were adjusted for known modifiers of the primary outcome, and are detailed in the results section. Post-transplant characteristics were analyzed as time-dependent covariates. A two-sided p value of less than 0.05 was considered to indicate statistical significance. Statistical analyses were performed with SAS 9.2 (SAS Institute, Cary, NC).

## Results

Overall, baseline samples were available for full-panel immunologic profiling from 69/89 (78%) HIV-HCV co-infected participants. The median (IQR) age of this subgroup was 49 (44–53), 51 (74%) were male, and 20 (29%) were black. The median (IQR) CD4+ T cell count was 286 (210–429) cells mm^-3^ and 6 (9%) had detectable HIV RNA. The median (IQR) follow-up time after transplant was 2.1 (0.7–4.0) years; 29 (42%) of participants died, 35 (51%) had graft loss, 14 (20%) had severe recurrent HCV, and 22 (32%) were treated for acute rejection (Table 1).

**Table 1:** Characteristics of HCV-HIV Coinfected Liver Transplant Recipients.

	HCV-HIV (N = 69)
**Recipient Characteristics**	
Age—years (median [IQR])	49 [44–53]
Male Gender—no. (%)	51 (74)
Black Race—no. (%)	20 (29)
BMI at Enrollment (median [IQR])	25 [23–29]
MELD at LT (median [IQR])	19 [14–26]
Hepatocellular Carcinoma—no. (%)	24 (35)
HCV Genotype 1/4 –no. (%)	56 (81)
HBV Co-Infection—no. (%)	56 (81)
CD4+ T cell (cells mm^-3^)^a^ –median [IQR]	286 [210–429]
CD4+% at LT—median [IQR]	24 [16–33]
HIV RNA detectable^a^ –no. (%)	6 (9)
**Donor/Transplant Characteristics**	
Donor Risk Index (DRI)–median [IQR]	1.36 [1.18–1.73]
Donor Age—years (median [IQR])	43 [25–50]
Black Donor Race—no. (%)	9 (13)
Anti-HCV Positive Donor—no. (%)	9 (13)
Combined Kidney-Liver Transplant—no. (%)	6 (9)
**Post-Transplant Characteristics**	
Follow-up Post-LT—yr (median [IQR])	2.1 [0.7–4.0]
Death—no. (%)	29 (42)
Graft Loss—no. (%)	35 (51)
Severe Recurrent HCV—no. (%)	14 (20)
Treated Acute Rejection—no. (%)	22 (32)

^a^ Most recent pre-transplant value, within 16 weeks of transplant.

LT—liver transplantation;

Markers of MT (LPS and sCD14) and monocyte activation (sCD163) were tested in samples pre-LT. There was little evidence of overt MT: only 8 (12%) of samples had detectable LPS using an optimized assay.[10] There appeared to be no demographic differences between subjects with and without MT (data not shown). Infrequent detection of LPS diminished the power for the evaluation of pre-LT LPS levels in predictive models of post-LT outcomes. Post-LT markers were not significantly different from pre-LT levels (data not shown).

sCD14 is released upon monocyte activation, and in particular in response to stimulation with LPS among other inflammatory stimuli. In contrast to infrequent LPS detection, sCD14 was robustly measured in all people. The median (IQR) of values was 3.48 (3.31–3.62) log_10_ pg mL^-1^, and was similar to what has been described previously.[7] Median (IQR) sCD14 level was 3.59 (3.43–3.65) log_10_ pg mL^-1^ in 8 subjects with detectable LPS and 3.48 (3.31–3.62) log_10_ pg mL^-1^ in 60 subjects with undetectable LPS (p = 0.27). This non-significant relationship was in contrast to what has been previously described, but may be due to the small numbers of samples with detectable LPS.[6, 7] In addition, pre-LT sCD14 levels were not associated with age (p = 0.12), but did appear to be marginally elevated in males (median of 3.52 vs. 3.35 log_10_ pg mL^-1^in females; p = 0.07) and significantly lower in blacks (median of 3.33 vs. 3.53 log_10_ pg mL^-1^ in non-blacks; p = 0.002).

**Fig 1:**
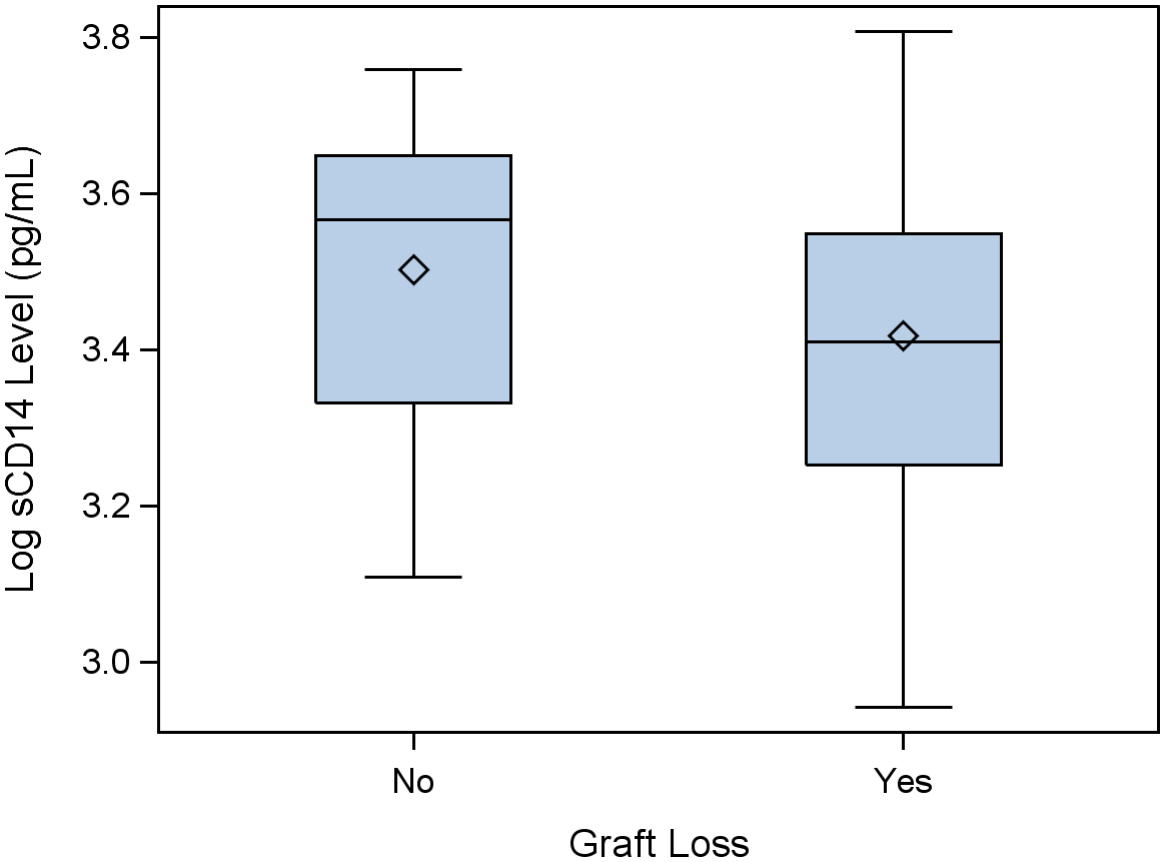
sCD14 Levels Pre-LT. sCD14 levels were measured by ELISA in 69 HIV-HCV co-infected persons pre-LT; graft loss post-LT occurred in 35/69 (51%) persons. In a univariate Cox regression model, higher sCD14 levels pre-LT were marginally associated with lower risk of graft loss post-LT (p = 0.08).

**Fig 2:**
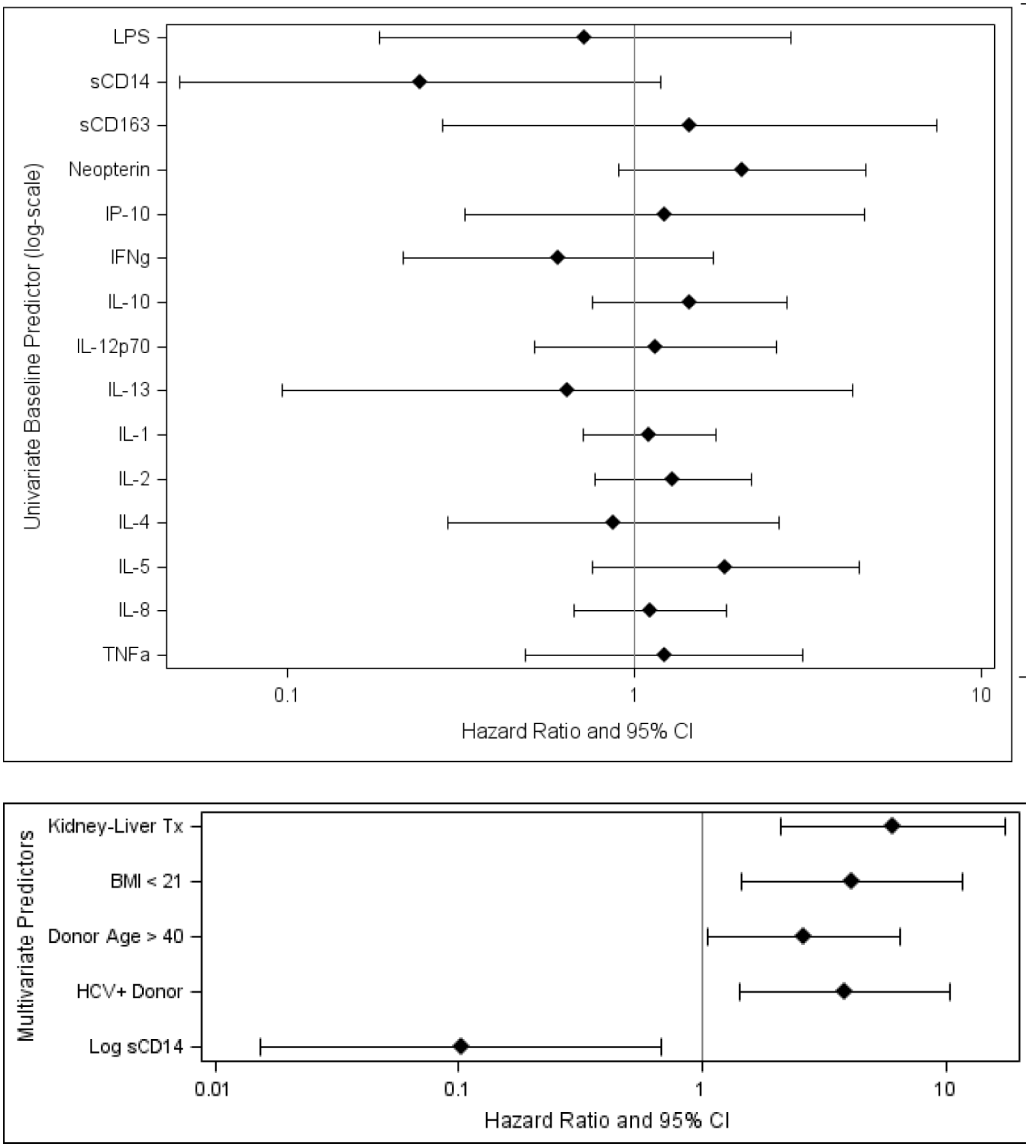
sCD14 pre-LT and Graft Loss. Proportional hazards regression models for graft loss were developed. Univariate analyses were performed with each biomarker singly (upper panel); multivariate models were developed that incorporated known predictors of graft loss and biomarkers found in the univariate analysis.

Higher sCD14 levels pre-LT were marginally associated with lower risk of graft loss post-LT in a univariate model (p = 0.08; Fig 1; S1 Table). In a multivariate model of graft loss, higher log_10_ sCD14 levels were associated with a 90% reduced risk of graft loss (p = 0.02; Fig 2; Table 2, after adjustment for donor age, BMI at enrollment, HCV donor status, and whether the participant had a combined kidney-liver transplant). Other markers of monocyte activation were also tested in baseline samples. Neopterin, a non-specific measure of inflammation, was found to be marginally associated with graft loss post-transplant, but lost its significance in a multivariate model that included sCD14 (data not shown). Surprisingly, sCD163 levels at baseline did not predict outcome and were not correlated with either sCD14 levels or neopterin levels.

**Table 2:** Univariate and Multivariate Proportional Hazards Regression Models for Graft Loss.

**Univariate Predictor** ^a^	**HR (95% CI)**	**P Value**
LPS	0.7 (0.2, 2.8)	0.64
sCD14	0.2 (0.05, 1.2)	0.08
sCD163	1.4 (0.3, 7.4)	0.67
Neopterin	2.0 (0.9, 4.6)	0.09
IP-10	1.2 (0.3, 4.6)	0.77
IFNγ	0.6 (0.2, 1.7)	0.33
IL-10	1.4 (0.8, 2.8)	0.26
IL-12p70	1.2 (0.5, 2.6)	0.73
IL-13	0.6 (0.1, 4.3)	0.64
IL-1	1.1 (0.7, 1.7)	0.67
IL-2	1.3 (0.8, 2.2)	0.33
IL-4	0.9 (0.3, 2.6)	0.80
IL-5	1.8 (0.8, 4.4)	0.18
IL-8	1.1 (0.7, 1.8)	0.69
TNFα	1.2 (0.5, 3.1)	0.68
**Multivariate Predictors** ^b^	**HR (95% CI)**	**P Value**
Kidney-Liver Tx	6.1 (2.1, 17.5)	0.001
BMI < 21	4.1 (1.5, 11.8)	0.01
Donor Age > 40	2.6 (1.1, 6.4)	0.04
HCV+ Donor	3.9 (1.4, 10.4)	0.01
Log sCD14	0.1 (0.02, 0.7)	0.02

Abbreviation: HR, Hazard Ratio

^a^ Baseline predictor (log-scale)

^b^ Incorporated known predictors of graft loss and biomarkers found in the univariate analysis.

**Fig 3:**
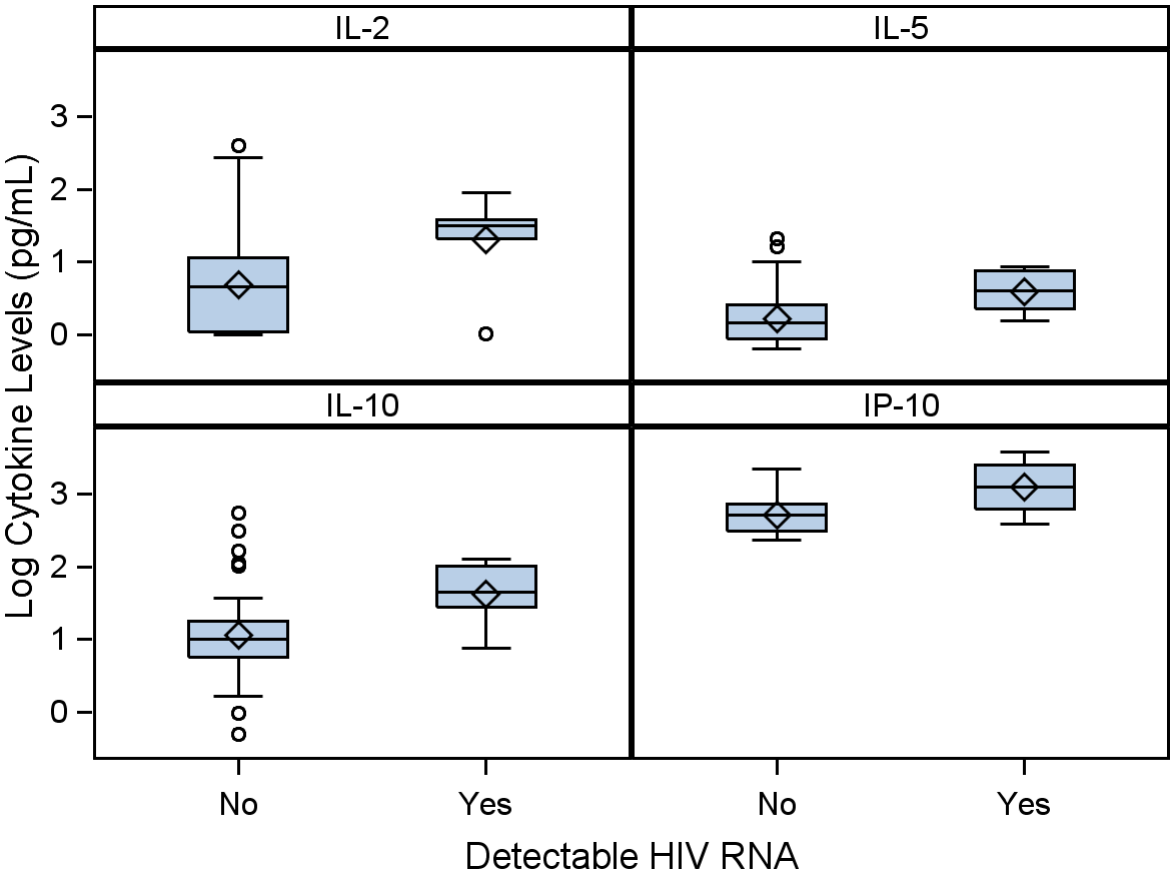
HIV RNA Levels Pre-LT and Immune Activation. Full immunologic profile was performed using the MSD platform, while IP-10 levels were measured by ELISA. Persons with detectable HIV RNA pre-LT had higher levels of IL-2 (p = 0.01), IL-5 (p = 0.02), IL-10 (p = 0.01), and IP-10 (p = 0.01), by Wilcoxon rank-sum test.

Transplant rejection results in part from the balance between Th1 and Th2 immunity; the former promotes rejection while the latter fosters tolerance to the allogeneic organ.[12, 13] To test the hypothesis that HIV infection leads to increased Th1 versus Th2 immunity, a panel of cytokines that broadly represent both limbs of cell-mediated immunity were measured pre-transplant. IL-10, a prototypical Th2 cytokine, was found to be significantly higher in persons with detectable HIV RNA at baseline compared to persons with suppressed HIV RNA (p = 0.01; Fig 3; S1 Table). Interferon-γ (IFNγ), the hallmark of Th1 immunity, contrastingly did not show a significant difference between persons with detectable and undetectable HIV RNA at baseline, although IP-10 (p = 0.01), IL-2 (p = 0.01), and IL-5 (p = 0.02) were significantly different between the groups.

**Fig 4:**
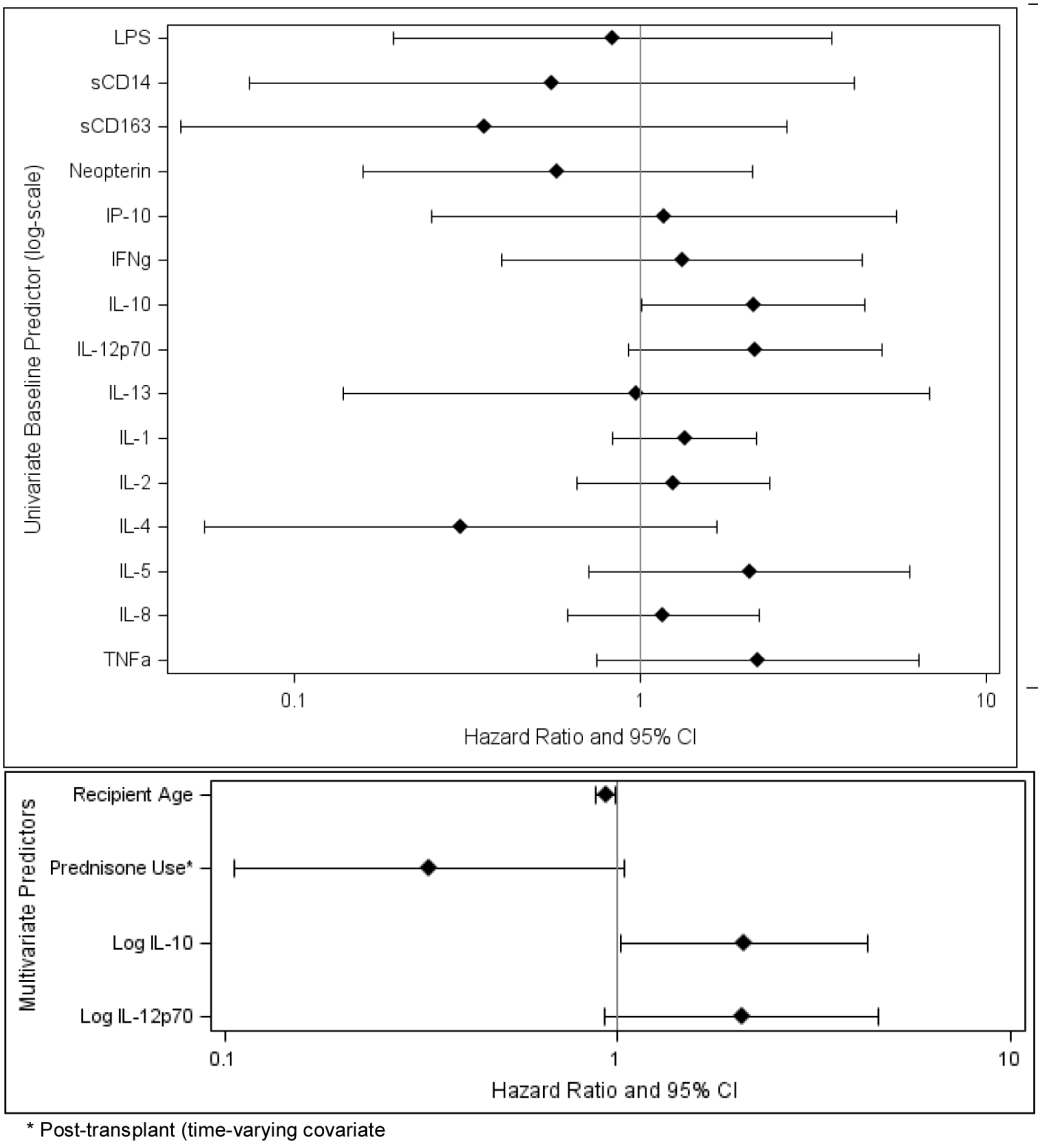
Proportional Hazards Regression Models for Treated Acute Rejection. Univariate analyses were performed with each biomarker singly (upper panel); multivariate models were developed that incorporated known predictors of treated acute rejection and biomarkers found in the univariate analysis.

In separate univariate models, only pre-LT IL-10 levels (p = 0.047) and IL-12p70 levels (p = 0.08) were predictive of acute rejection (Fig 4; Table 3). In a multivariate model of treatment for acute rejection that was adjusted for recipient age and prednisone use, pre-LT IL-10 was the only cytokine that was significantly associated with outcome: higher log_10_ IL-10 levels were associated with a two-fold increased risk of acute rejection (HR = 2.10, 95% CI 1.01–4.34, p = 0.046).

**Table 3:** Univariate and Multivariate Proportional Hazards Regression Models for Treated Acute Rejection.

**Univariate Predictor** ^a^	**HR (95% CI)**	**P Value**
LPS	0.8 (0.2, 3.6)	0.80
sCD14	0.6 (0.1, 4.2)	0.57
sCD163	0.4 (0.05, 2.7)	0.31
Neopterin	0.6 (0.2, 2.1)	0.40
IP-10	1.2 (0.3, 5.5)	0.84
IFNγ	1.3 (0.4, 4.4)	0.65
IL-10	2.1 (1.0, 4.5)	0.047
IL-12p70	2.1 (0.9, 5.0)	0.08
IL-13	1.0 (0.1, 6.9)	0.98
IL-1	1.3 (0.8, 2.2)	0.23
IL-2	1.3 (0.7, 2.4)	0.50
IL-4	0.3 (0.1,1.7)	0.17
IL-5	2.1 (0.7, 6.0)	0.18
IL-8	1.2 (0.6, 2.2)	0.64
TNFα	2.2 (0.7, 6.4)	0.16
**Multivariate Predictors** ^b^	**HR (95% CI)**	**P Value**
Recipient Age	0.93 (0.88, 0.98)	0.01
Prednisone Use^c^	0.3 (0.1, 1.04)	0.06
Log IL-10	2.1 (1.01, 4.3)	0.046
Log IL-12p70	2.1 (0.9, 4.6)	0.08

Abbreviation: HR, Hazard Ratio

^a^ Baseline predictor (log-scale)

^b^ Incorporated known predictors of treated acute rejection and biomarkers found in the univariate analysis.

^c^ Post-transplant (time-varying covariate)

Although nearly every transplanted liver is re-infected with HCV in persons with chronic infection, a subset of persons develop severe recurrence of HCV that results in rapid onset cirrhosis, requiring repeat liver transplant. None of the tested biomarkers appeared to predict severe HCV recurrence (data not shown).

## Discussion

While newer, highly effective direct acting antivirals (DAA) for HCV in the peri-transplant period have become available, there are still challenges in the co-administration of DAA with HAART and immunosuppressive medicines required for LT. In addition, curing patients of HCV prior to LT may limit their access to some organs. Therefore, it has become even more important to stratify HIV-HCV co-infected persons on the basis of their risk for developing poor outcomes post-LT. The present study is a first demonstration of immunologic biomarkers that predict post-LT outcomes in HIV-HCV co-infection. Herein, we have shown that elevated sCD14 levels pre-LT were strongly predictive of graft survival post-LT, while high IL-10 levels pre-LT were predictive of the need for treating acute rejection post-LT. These results suggest that a tipped immunologic balance toward immune activation pre-LT, rather than immune tolerance, is associated with improved health of the transplanted liver.

To date, there have been several large observational studies of LT in HIV-HCV co-infection in the post-HAART era. Terrault et al., using the HIVTR data, found that major predictors of graft loss post-LT were low BMI in the recipient, older age and HCV+ in the donor, and combined liver-kidney transplantation.[5] Moreover, HIV-HCV co-infected persons were more likely to require treatment for acute rejection than HCV mono-infected persons. Similarly, Miro et al. found that HIV-HCV co-infected persons had nearly a two-fold higher risk of needing treatment for acute rejection than HCV mono-infected persons, and had lower rates of graft survival.[14] Earlier studies noted higher mortality post-LT in HIV infection, but the causes of death were largely attributable to HIV-related infections, especially since these patients were not receiving suppressive HAART.[15] There have been few studies that have uncovered the biological underpinnings of the poorer outcomes found in HIV. The present study is based on the HIVTR study of 89 HIV/HCV co-infected persons who underwent liver transplantation in 17 participating centers from October, 2003 to February, 2010, which represents approximately 50–65% of all such transplants that took place nationally during the same accrual period. Therefore, our investigation is likely to be broadly representative of HIV/HCV co-infected persons undergoing liver transplantation in the US.

The present study revealed novel findings that lead to further questions. sCD14 is a marker of monocyte, macrophage, and hepatocyte activation and has been associated with HIV progression and cirrhosis in non-transplantation cohorts of HIV mono-infected and HIV-HCV co-infected persons, respectively.[6, 7] Elevated sCD14 levels have been found to be associated MT and are predictive of hepatic fibrosis in chronic viral hepatitis.[6, 7, 16–19] Intriguingly, in an African cohort of persons in whom liver disease was documented without another identified cause, sCD14 appeared to have opposite effects depending on HIV status: among HIV uninfected persons, higher sCD14 levels were associated with lower liver stiffness, whereas among HIV infected persons, lower sCD14 levels were associated with lower liver stiffness.[20] Consistent with the latter study’s findings, we found that elevated sCD14 levels pre-LT were associated with graft survival post-LT. Taken together, it is possible that innate immune activation pre-LT may be protective for the nascent graft when HIV is controlled. It is also possible that pre-LT sCD14 levels reflect MT that was not detected by the adapted LPS assay; since MT products are poorly cleared by the impaired pre-LT liver, high pre-LT sCD14 levels may have identified persons who benefitted most from LT. The role of innate immune activation, and sCD14 levels, in HIV-HCV LT and liver disease progression will need further study and validation.

IL-10 is a counter-regulatory cytokine that is secreted in the context of Th2 immunity, and is frequently elevated in progressive hepatic fibrosis.[21–24] The association between IL-10 levels and acute rejection suggests that Th2 tolerance in advanced liver disease pre-LT may paradoxically promote rejection post-LT. It is possible that the latter association is concordant with the production of anti-allograft antibodies that results from Th2 skewing.[25] It is interesting to note that prednisone use was marginally associated with decreased treatment for graft rejection, although it is possible that more exposure to corticosteroids decreased the risk of rejection.

It is unclear how interferon-free combination DAA treatment of HCV will impact the immunologic associations that we report here. Previously, liver transplant candidates with decompensated cirrhosis were not able to receive interferon-based therapy because of the risk of further decompensation. Recent studies have shown promising rates of sustained virologic response (SVR) and improvements in MELD scores for persons with HCV infection and decompensated cirrhosis [26], and it is possible that this will translate to a decreased need for liver transplantation in some patients. Indeed, SVR alone has been associated with a marked improvement in liver-related mortality among HIV/HCV co-infected persons, even among persons with advanced liver disease.[27] However, further investigation is required to determine whether SVR pre-transplant will impact the likelihood of post-transplant graft loss and/or rejection.

The current study has several limitations. Serum levels of cytokines may not reflect tissue levels, which are likely concentrated at the site of action to induce more direct effects; therefore, recipient liver biopsies immediately post-LT may be more informative of LT outcomes. In addition, cellular immune responses were not directly measured due to a lack of availability of PBMCs. A further challenge was in the measurement of MT: to adapt the LPS detection assay to human serum required dilution of samples; therefore, it is possible that MT occurred in some participants but was below the level of sensitivity of our assay. A fourth limitation is that the cohort was largely white and male; therefore, the principal findings in this report will need validation in other cohorts before generalizing the key biomarkers to all HIV-HCV co-infected persons undergoing LT.

In summary, we found evidence that pre-LT monocyte activation was predictive of protection against post-LT graft loss in a well-characterized registry of HIV-HCV co-infected persons. Moreover, we found that high IL-10 levels pre-LT was associated with treatment for acute rejection post-LT. Taken together, these results suggest that skewing of multiple arms of immunity toward a pro-inflammatory state pre-LT may be counter-intuitively protective post-LT, and may help stratify HIV-HCV co-infected patients undergoing LT who may require additional vigilance after transplantation. Future research is needed to understand the mechanisms underlying the protective effect of immune activation in LT, and to target modifiable pathways that could improve LT outcomes in HIV-HCV co-infection.

## Supporting Information

S1 Table Supplementary Information Minimal Anonymous Dataset. The supporting data set includes all of the data required to generate the principal findings for Figs 1 and 3.
(XLS)
